# Identification of sarcomatoid differentiation in renal cell carcinoma by machine learning on multiparametric MRI

**DOI:** 10.1038/s41598-021-83271-4

**Published:** 2021-02-15

**Authors:** Asim Mazin, Samuel H. Hawkins, Olya Stringfield, Jasreman Dhillon, Brandon J. Manley, Daniel K. Jeong, Natarajan Raghunand

**Affiliations:** 1 Department of Cancer Physiology, Moffitt Cancer Center, Tampa, FL 33612 USA; 2 IRAT Shared Service, Moffitt Cancer Center, Tampa, FL 33612 USA; 3 Department of Anatomic Pathology, Moffitt Cancer Center, Tampa, FL 33612 USA; 4 Department of Genitourinary Oncology, Moffitt Cancer Center, Tampa, FL 33612 USA; 5 Department of Diagnostic & Interventional Radiology, Moffitt Cancer Center, Tampa, FL 33612 USA; 6 Department of Oncologic Sciences, University of South Florida, Tampa, FL USA; 7 Present Address: Department of Computer Science & Information Systems, Bradley University, Peoria, IL 61625 USA

**Keywords:** Diagnostic markers, Cancer imaging, Renal cancer

## Abstract

Sarcomatoid differentiation in RCC (sRCC) is associated with a poor prognosis, necessitating more aggressive management than RCC without sarcomatoid components (nsRCC). Since suspected renal cell carcinoma (RCC) tumors are not routinely biopsied for histologic evaluation, there is a clinical need for a non-invasive method to detect sarcomatoid differentiation pre-operatively. We utilized unsupervised self-organizing map (SOM) and supervised Learning Vector Quantizer (LVQ) machine learning to classify RCC tumors on T2-weighted, non-contrast T1-weighted fat-saturated, contrast-enhanced arterial-phase T1-weighted fat-saturated, and contrast-enhanced venous-phase T1-weighted fat-saturated MRI images. The SOM was trained on 8 nsRCC and 8 sRCC tumors, and used to compute Activation Maps for each training, validation (3 nsRCC and 3 sRCC), and test (5 nsRCC and 5 sRCC) tumor. The LVQ classifier was trained and optimized on Activation Maps from the 22 training and validation cohort tumors, and tested on Activation Maps of the 10 unseen test tumors. In this preliminary study, the SOM-LVQ model achieved a hold-out testing accuracy of 70% in the task of identifying sarcomatoid differentiation in RCC on standard multiparameter MRI (mpMRI) images. We have demonstrated a combined SOM-LVQ machine learning approach that is suitable for analysis of limited mpMRI datasets for the task of differential diagnosis.

## Introduction

Sarcomatoid differentiation in renal cell carcinoma (sRCC) is histologically characterized by anaplastic changes of renal cell carcinoma (RCC) subtypes and is associated with a poorer prognosis than RCC without sarcomatoid components (nsRCC) ^1^. In a retrospective analysis of 27,856 subjects with RCC, Liu et al. concluded that presence of a sarcomatoid component was associated with poor overall survival (Hazard ratio 1.89) and shorter progression-free survival (Hazard ratio 2.04) ^2^. Age at diagnosis, T stage, N stage, presence or absence of metastases to bone, liver or lung, and nephrectomy have been reported to be independent predictors for overall survival in sRCC ^3^. sRCC is managed more aggressively than nsRCC, and there is a clinical need for a non-invasive method to detect sarcomatoid differentiation pre-operatively, especially when considering management options like active surveillance ^4^.

Artificial intelligence (AI) is a new paradigm in medical imaging that promises greater productivity, efficiency, and accuracy in the practice of Radiology ^5^. Two AI streams are widely adopted in the field of radiological imaging. The first stream utilizes machine learning on handcrafted features extracted from the image data ^6^, while the second approach uses deep learning algorithms such as Convolutional Neural Networks (CNN) that “learn” patterns directly from large amounts of training data ^7^. Both approaches have been leveraged for the task of differential diagnosis to classify images acquired with various modalities such as magnetic resonance imaging (MRI) and computed tomography (CT) for a variety of pathologies ^6,8,9^. Deep learning techniques have gained considerable attention in the medical image analysis domain with encouraging results on diverse applications, such as differential diagnosis of Alzheimer’s Disease vs. Mild Cognitive Impairment ^10^, thyroid nodule classification on ultrasound ^11^, differentiation of liver masses on dynamic contrast-enhanced CT ^12^, and staging of liver fibrosis on gadoxetic acid-enhanced liver MRI ^13^. Image classification is a core machine learning application that involves differentiation between pre-established categories, such as normal versus pathological tissue types. For example, Chan et al. used a statistical classifier to detect prostate cancer by combining information from MR images and employing a support vector machine (SVM) on handcrafted features to predict the likelihood of tumor presence in the peripheral zone of the prostate gland ^14^. Litjens et al. used a random forest classifier on individual and combined multiparameter MRI images to generate a probabilistic map of cancer location in the prostate ^15^.

In this work we have used the terms “training”, “validation”, and “testing” as recommended by Park and Han for the use of AI in for medical diagnosis and prediction ^16^. Training is the process of using a set of observations to generate a model. Validation is an initial, unblinded, assessment of the generalizability of the trained model, and it is common to loop between the training and validation steps numerous times to refine the model and achieve acceptable performance on the validation dataset. Testing refers to the assessment of the performance of a single model, finalized at the validation stage, on an unseen (or held-out) dataset. If the model performance on testing is unsatisfactory, revision of the model and retesting on the same cohort is not permitted. Rather, the previous test data must be relegated to the training and validation data, and new unseen test data must be found for subsequent testing of an improved model ^17^.

Studies that follow the complete training-validation-testing paradigm for the task of differential diagnosis of RCC on radiologic images are uncommon in the literature (reviewed in ^18^). In an analysis of voxel-based whole lesion enhancement on MRI, Chandarana et al. identified histogram parameters that could discriminate between clear cell and papillary subtypes of renal cell cancer with an accuracy of 94.6%, sensitivity of 96%, and specificity of 90%, on a training dataset of 19 papillary RCCs and 55 clear cell RCCs ^19^. Varghese et al. reported a CT texture feature model that achieved an area under the receiver operating characteristic curve of 0.87 for differentiating benign from malignant solid enhancing lipid-poor renal masses on training data that comprised 45 benign renal masses and 129 malignant RCC tumors of clear cell, papillary and chromophobe subtypes ^20^. Kocak et al. ^21^ utilized artificial neural networks (ANNs), support vector machines, and their enhanced variations, to classify RCC subtypes based on texture features extracted from unenhanced and corticomedullary phase contrast-enhanced CT images. They investigated 4 SVM and 4 ANN models for the task of classifying clear cell RCC (ccRCC) from non-ccRCCs and achieved validation accuracies of 64.6–84.9%. They also reported validation accuracies between 57.6 and 69.2% for the task of classifying individual RCC subtypes (clear cell vs. papillary cell vs. chromophobe cell RCC). Zabihollahy et al. ^22^ used semi-automated majority voting 2D-CNN, fully automated 2D-CNN, and 3D-CNN to classify RCC from benign solid renal masses on contrast-enhanced computed tomography (CECT) images, and achieved validation accuracies of 77.36–83.75%. In a study of 38 low-grade and 11 high-grade ccRCC tumors < 4 cm, Schieda et al. ^23^ investigated the utility of standard pre-operative MRI to differentiate low-grade from high-grade clinical T1a ccRCC. They evaluated subjective features including tumor size, as well as apparent diffusion coefficient (ADC) histogram analysis, contrast enhancement wash-in and wash-out rates, and a chemical shift signal intensity index related to water/fat content. Corticomedullary phase contrast wash-in rate, a chemical shift signal intensity index related to microscopic fat content in the tumor, and tenth-centile ADC, were noted to be higher in low-grade compared to high-grade clinical T1a ccRCC. A logistic regression model that combined these features produced an accuracy of 98% with a sensitivity of 87.5% and specificity of 100% on their training cohort.

In the present study we have sought to develop a machine learning model for the task of differential diagnosis of sRCC vs. nsRCC using balanced and tumor volume-matched training, validation and test cohorts. We report a combined unsupervised self-organizing map (SOM) and supervised Learning Vector Quantizer (LVQ) machine learning approach to classify RCC tumors based on their appearance on T2-weighted (T2W), non-contrast T1-weighted fat-saturated (T1W), contrast-enhanced arterial phase T1-weighted fat-saturated (T1W-CEart), and contrast-enhanced venous phase T1-weighted fat-saturated (T1W-CEven) MRI images.

The Kohonen self-organizing map (SOM) is an unsupervised neural network method for mapping high-dimensional data onto a regular low-dimensional grid, commonly a two-dimensional grid, on which nodes that describe more similar data points are closer to each other than nodes that describe more dissimilar data elements ^24^_._ The node or “neuron” that is most similar to an input is called the best matching unit (BMU), and while BMU can function as a simple classifier ^25^, in general SOM is not a classifier but rather provides an excellent way to visualize high-dimensional data. For example, Nattkemper and Wismuller ^26^ have mapped six-dimensional signal features extracted from ROIs in dynamic contrast-enhanced magnetic resonance imaging (DCE MRI) onto a two-dimensional space using SOM and used the SOM-based visualization to classify tumor pixels. The neurons on a trained SOM represent discrete locations or bins in the continuous multidimensional image intensity input space, to which pixels in a test tumor can be assigned based on closest Euclidean distance to produce hit maps or “Activation Maps”. Such Activation Maps are suitable for follow-on analyses using supervised learning algorithms. SOM analysis has been utilized to enable classification of mouse tumors on MRI images, and for classification of subcellular localization, mitotic phases and discrimination of apoptosis in fluorescence microscopy images of plant and human cells ^27^. In a multiparameter diffusion tensor MRI study of glioma, Inano et al. first used an SOM to group the multidimensional voxel intensities, then used K-means clustering on the SOM outputs to create cluster maps of the images, which were then classified using a Support Vector Machine (SVM) for distinguishing between low-grade and high-grade gliomas ^28^. Singh and Samavedham used SOM followed by SVM for differential diagnosis of early stage Parkinson’s disease (PD) vs. subjects without evidence of dopaminergic deficit (SWEDD) and healthy controls on T1-weighted MRI images ^29^. Alirezaie used the Learning Vector Quantizer (LVQ) neural network to classify and segment tissues in multiparameter MRI images of the brain using pixel intensity values ^30^.

An attraction of Activation Maps is that they are readily amenable to visual assessment, affording the possibility of simultaneous interpretation of the dimensionality-reduced image data depicted as 2D Activation Maps by both a machine learning algorithm and the human expert (i.e., Radiologist). Combined SOM-LVQ analysis may also require less training data than deep neural network methods, though at the expense of needing greater pre-processing of the input data. In the current study we have used SOM analysis to reduce the pixelwise information in co-registered and intensity-calibrated T2W, T1W, T1W-CEart and T1W-CEven MRI images, to produce 2-dimensional Activation Maps that were then used as inputs to train a supervised LVQ classifier for the task of identifying sarcomatoid differentiation in renal cell carcinoma on an unseen test dataset.

## Methods

We have investigated a combined unsupervised and supervised machine learning approach to classify RCC tumors based on their appearance on mpMRI images. In “Study subjects and MRI protocols” section we describe the retrospective accrual of human subjects and the MRI imaging. In “Image pre-processing” section we describe the pre-processing of the mpMRI images, which entailed (i) the pixel-level spatial alignment of the four MRI image sets to each other, (ii) followed by manual delineation of the tumors and semi-automatic delineation of the contralateral renal cortex, and, (iii) a method to calibrate the four MRI image sets so as to make pixel intensities on a given scan type comparable across subjects. In “Self-organizing map and activation maps” section we describe the process for training the SOM and reducing the four 3D MRI image sets of a given tumor to a single 2D “Activation Map” that serves as a “fingerprint” of that tumor. In “Learning vector quantization classifier (LVQ)” section we describe the process for training an LVQ model to classify tumors as sRCC or nsRCC based on their Activation Maps.

### Study subjects and MRI protocols

This retrospective study was approved by the Institutional Review Board (IRB) of the University of South Florida, Tampa, Florida, USA, which also waived the requirement for informed consent since human subjects data were collected retrospectively and analyzed after de-identification. All human subjects research was conducted in accordance with relevant institutional and national guidelines, including the US Health Insurance Portability and Accountability Act (HIPAA). In this study we identified 32 subjects belonging to the two classes nsRCC and sRCC that were matched for tumor volumes and sub-divided into three cohorts for model training, validation and testing, as in Table 1. Due to the limited sample sizes, the training cohort was augmented during certain steps to include a 1.6 cm^3^ sarcomatoid urothelial-origin kidney tumor and a volume-matched 1.4 cm^3^ non-sarcomatoid clear cell RCC tumor.

**Table 1:** Training, validation and test cohorts for SOM and LVQ analyses.

Cohort		Tumor class non-sarcomatoid	Tumor volume, cm^3^	Tumor class sarcomatoid	Tumor volume, cm^3^
SOM Phase	LVQ Phase				
Training	Training	Clear cell RCC	15.4	Clear cell RCC	13.7
		Clear cell RCC	55.6	Clear cell RCC	106.6
		Clear cell RCC	196.6	Clear cell RCC	187.9
		Clear cell RCC	306.4	Clear cell RCC	305.9
		Clear cell RCC	501.8	Chromophobe RCC	459.7
		Clear cell RCC	610.8	Clear cell RCC	611.0
		Clear cell RCC	1135.0	Clear cell RCC	731.1
		Clear cell RCC	1425.0	Clear cell RCC	953.5
Not used	Validation	Clear cell RCC	410.3	Clear cell RCC	442.9
		Clear cell RCC	683.8	Clear cell RCC	866.4
		Clear cell RCC	1184.7	Papillary RCC	1440.7
Not used	Testing	Clear cell RCC	175.2	Clear cell RCC	182.0
		Clear cell RCC	263.3	Clear cell RCC	269.3
		Clear cell RCC	380.6	Clear cell RCC	372.6
		Clear cell RCC	509.0	Chromophobe RCC	490.9
		Clear cell RCC	1258.8	Clear cell RCC	1291.1

Pre-operative MRI scans of all subjects were acquired at 1.5 T on scanners manufactured by Siemens (Siemens Healthineers, Erlangen, Germany), GE (GE Healthcare, Chicago, IL), or Toshiba (Canon Medical Systems USA, Tustin, CA). T2W images were acquired using single-shot fast spin echo sequences (HASTE/SS-FSE/FASE), and T1W images were acquired using spoiled gradient echo sequences. Contrast media used in these studies was gadobutrol (Bayer, Whippany, NJ, USA) administered intravenously at 0.1 mL/kg body weight. Gadobutrol was injected at 1.5 mL/s followed by 35 mL Normal Saline. Arterial phase T1-weighted imaging was performed 30 s following contrast injection, and venous phase T1-weighted imaging was performed 90 s post-injection.

### Image pre-processing

**Figure 1:**
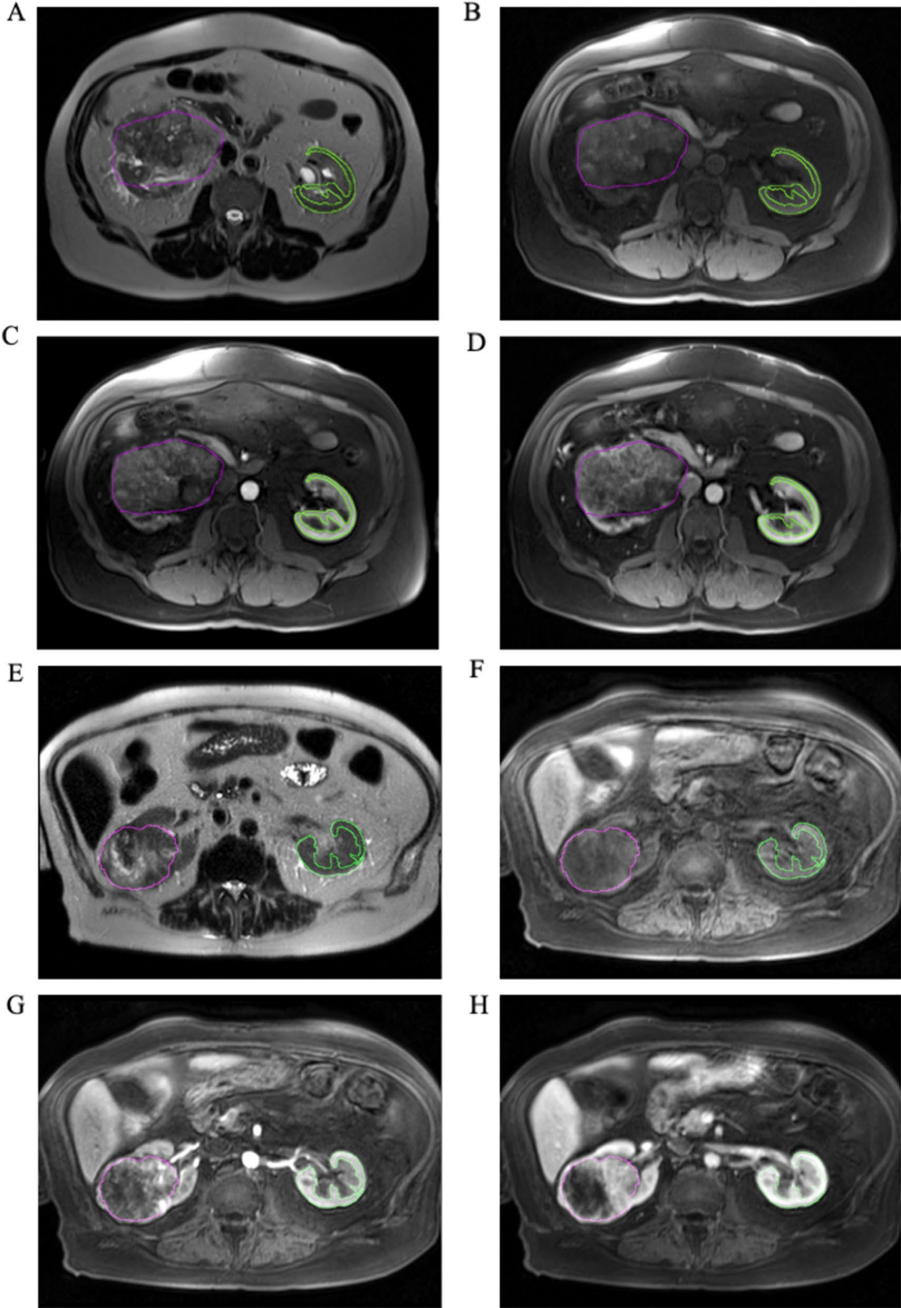
Example axial mpMRI images of two subjects, one with an nsRCC tumor (contoured in purple in (**A**–**D**) and another with an sRCC tumor (contoured in purple in (**E**–**H**)). (**A**,**E**) T2W; (**B**,**F**) non-contrast T1W fat-saturated; (**C**,**G**) contrast-enhanced T1W fat-saturated arterial-phase; (**D**,**H**) contrast-enhanced T1W fat-saturated venous-phase images. The semi-automatically drawn contours of the contralateral normal renal cortex are shown in green in all panels.

For each subject, tumors were segmented by manual contouring on all applicable slices of the axial T2W scan by an experienced Radiologist (DKJ). Next, the T1W, T1W-CEart and T1W-CEven images were resampled and spatially co-registered to the T2W image for each subject using in-house MATLAB (MathWorks, Natick, MA) software as previously described ^4,31^. Global rigid registration was performed to correct for gross differences in slice planning between sequences, followed by local affine registration to achieve fine co-registration between the sequences in a volume-of-interest around the tumor. Following co-registration, the contralateral normal kidney cortex was semi-automatically segmented on all applicable slices of the T1W-CEart images using the “magic wand” function of the ImageJ software (imagej.net), and the resulting mask was applied to the other 3 co-registered MRI sequences as shown in Fig. 1.

Due to the retrospective nature of this study, the MRI acquisition parameters varied from subject to subject, which we accounted for by calibration of pixel intensities as follows. Raw tumor voxel intensities on T2W images were calibrated by dividing them by the mean intensity of the contralateral normal kidney cortex on T2W of the same subject. Raw tumor voxel intensities on T1W, T1W-CEart and T1W-CEven images were calibrated by dividing them by the mean intensity of the contralateral normal kidney cortex on unenhanced T1W of the same subject. Calibrated voxel intensities from tumors belonging to the training and validation cohorts were variance-normalized to the means and standard deviations of pooled training + validation tumor voxels on each respective scan type; these same means and standard deviations were then used to variance-normalize intensities of voxels in tumors from the test cohort.

### Self-organizing map and activation maps

After calibration and variance-normalization each voxel had 4 “channels” of mpMRI intensities associated with it ^32–34^. We reasoned that each mpMRI “channel” may be adequately described by 3 intensity levels (low, medium, high), for a total of 3^4^ = 81 levels across the 4 channels. We therefore computed a 9 $\times$ 9 Kohonen Self-Organizing Map ^24^ on the 18 training cohort tumors using the MiniSom ^35^ library. The SOM was trained using the following parameters: a lattice size of 9 $\times$ 9, “bubble function” as the neighborhood function, a learning rate of 0.2, and 1,000,000 iterations. The reader is referred elsewhere ^24^ for a detailed understanding of the process of training a SOM. In our case the output at the end of the SOM training process was a 9 $\times$ 9 map of 81 “neurons”, with each neuron representing a discrete location in the 4-dimensional calibrated and variance-normalized continuous mpMRI intensity space. Thus, each neuron represents a 4-parameter mpMRI “phenotype” to which a given voxel in a test tumor could potentially belong. By assigning each voxel in a given tumor to the neuron in the trained SOM having closest Euclidean proximity, 34 “Activation Maps” were generated from the 34 training (augmented), validation and test tumors in our study.

### Learning vector quantization classifier (LVQ)

**Figure 2:**
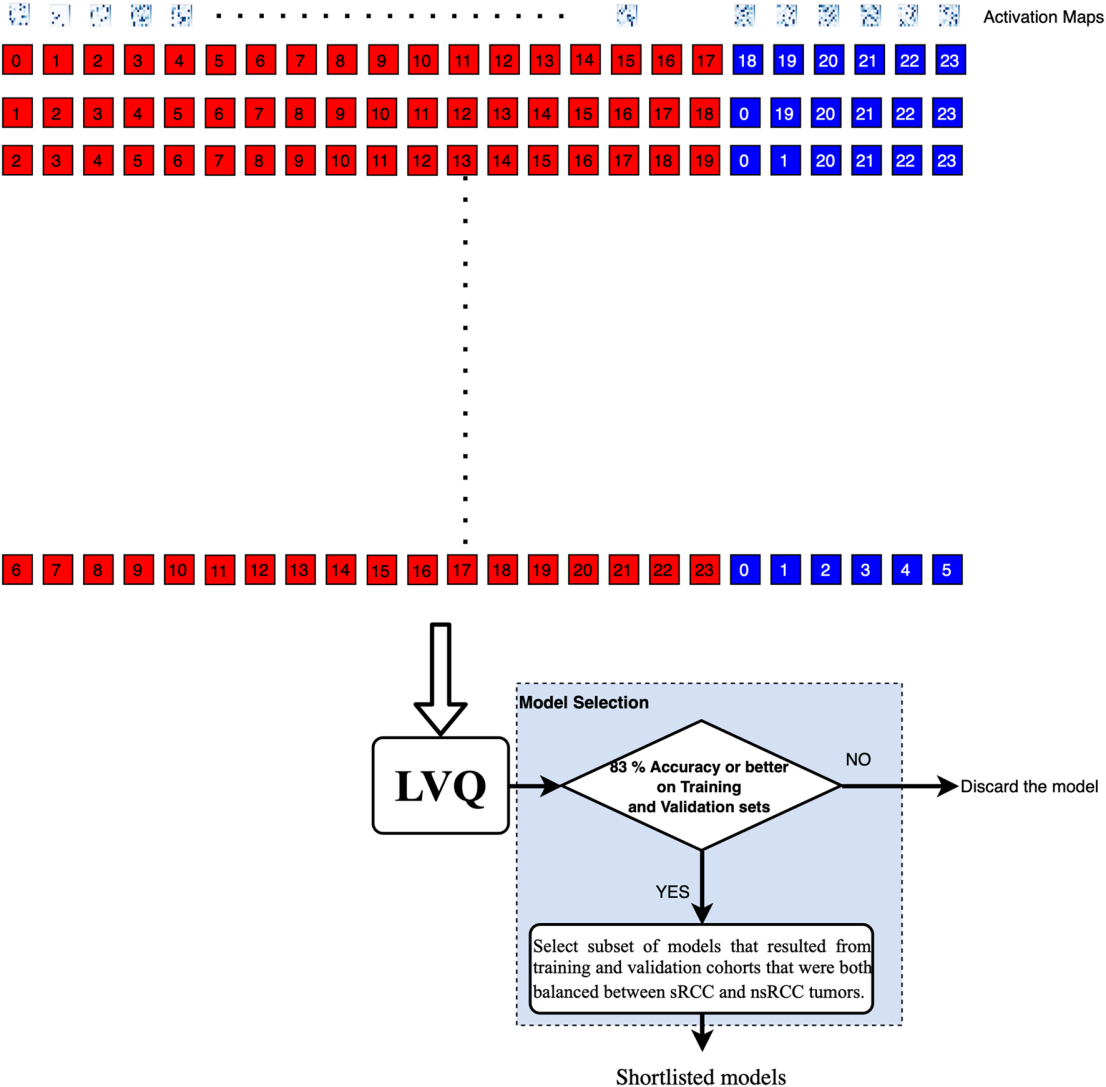
Cross-validation and model selection process.

The LVQ is a supervised learning method for defining classes in the input space that uses class information (sRCC or nsRCC) to move decision boundaries known as Voronoi cells to maximize classifier performance ^36^. The inputs for training the LVQ were vectors of dimension 81 $\times$ 1 that were obtained by reshaping the 9 $\times$ 9 Activation Maps from each of the 18 tumors in the augmented training cohort (Table 1). Trained LVQ models were evaluated for generalizability by assessing their diagnostic performance on vectors of dimension 81 $\times$ 1 corresponding to the 9 $\times$ 9 Activation Maps from each of the 6 tumors in the validation cohort (Table 1). In addition to training and validation on separate cohorts in this manner (“simple validation”), we also explored leave-six-out cross-validation on the combined 24 training and validation tumors; this process is illustrated in Fig. 2.

The sample size for cross-validation comprised a total of 3,293,112 voxels from 24 tumors with known labels (sRCC or nsRCC). In the first phase of leave-six-out cross-validation there were 134,596 training-validation combinations, of which 8 combinations produced $\ge$ 83% accuracy on both the training and validation cohorts. Of these eight models, three models resulted from combinations of tumors that were balanced between the tumor classes in both the training and validation sets. All three of these final three models yielded validation accuracies that were identical to the performance of the model initially identified during “simple validation” on separate training and validation cohorts. We therefore decided to select the LVQ model from simple validation for further optimization; the SOM and the LVQ models were thus trained on exactly the same set of 18 tumors. The selected LVQ model was further optimized for learning rate and number of training epochs on the 6 validation tumors prior to testing on an independent set of 10 tumors (Table 1). Throughout the entire process we sought to match the distribution of tumor volumes between the nsRCC and sRCC classes in all three cohorts, on the hypothesis that this would minimize the influence of tumor volume as a confounding covariate.

## Results

### Activation maps from SOM analysis

**Figure 3:**
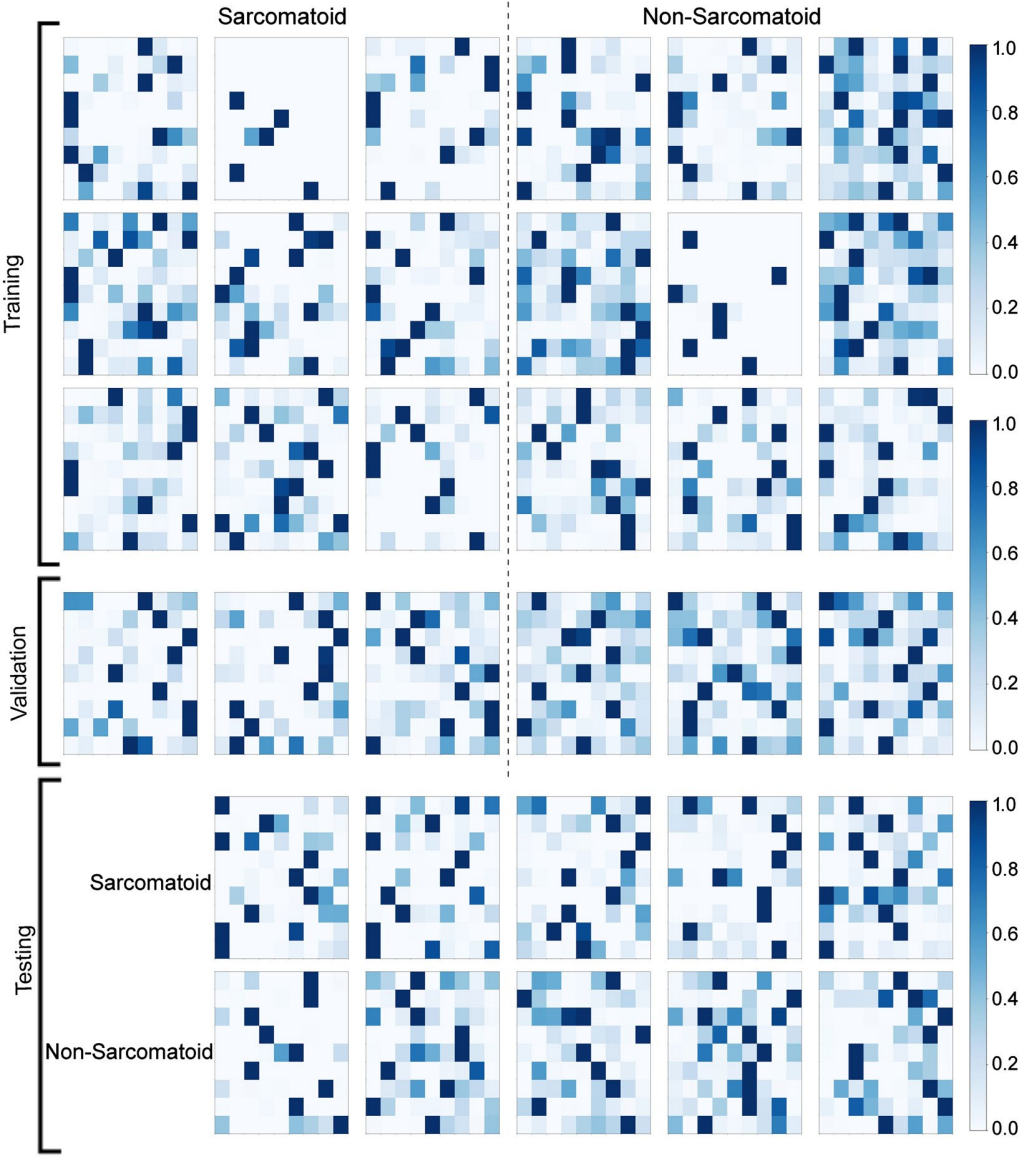
Activation maps for each tumor generated by assigning hits from every voxel in a given tumor to the 81 neurons in the trained SOM based on closest Euclidean proximity.

Every voxel in a given tumor was assigned to that neuron in the trained SOM to which it was closest in Euclidean proximity, after which the total number of “hits” per neuron was normalized between 0 and 1 and the results depicted as a 9 $\times$ 9 Activation Map for that tumor. Activation Maps corresponding to all 34 tumors in our analysis are shown in Fig. 3. Each neuron represents a unique mpMRI “phenotype”, making each Activation Map a 2D representation of that tumor’s composite mpMRI phenotype.

### Training and validation of the LVQ classifier

**Figure 4:**
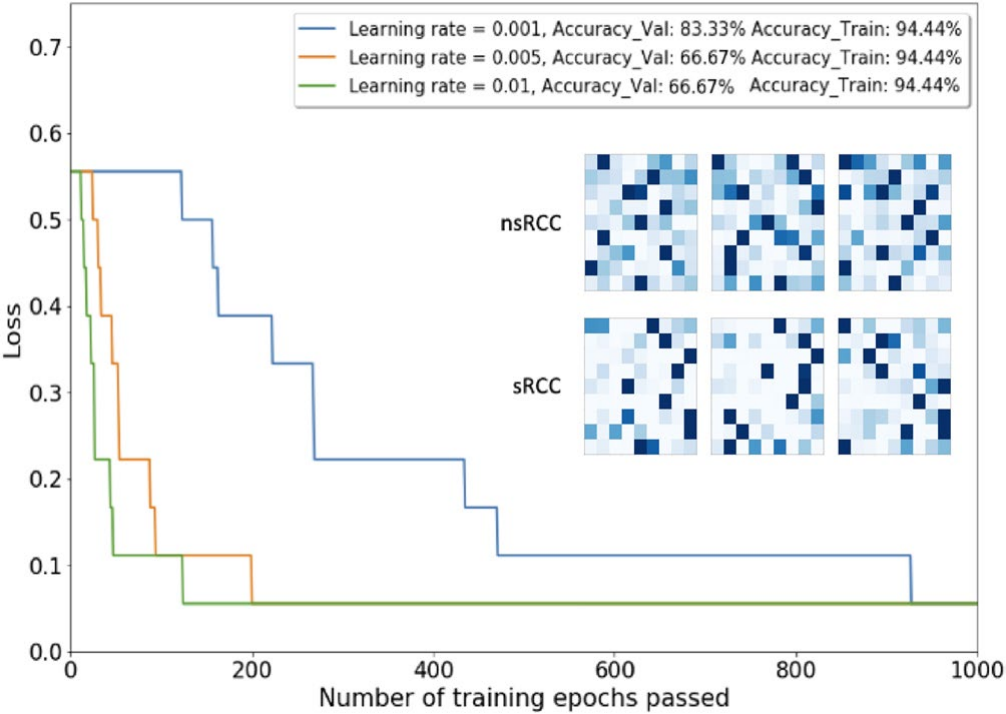
The main plot shows loss (misclassification ratio) as a function of step size (learning rate) and number of training epochs of LVQ model. The insert shows individual Activation Maps of the six Validation Cohort tumors.

The individual Activation Maps became inputs for training the LVQ classifier. The LVQ model selected after simple validation was further optimized for the learning rate and the number of training epochs to minimize the loss function versus the training epochs for three different learning rates as shown in Fig. 4. The model with 0.001 learning rate and 1000 training epochs achieved 83.33% accuracy on the validation cohort and 94.44% on the augmented training set as illustrated in Fig. 4. Following the complete training-validation-testing paradigm ^16,17^, this model was advanced to final testing on an unseen cohort of 5 nsRCC and 5 sRCC tumors.

### Testing of the LVQ classifier

On the test cohort the final model produced an overall accuracy of 70%, with a false negative rate of 20% for misclassifying the sRCC as nsRCC, and a false positive rate of 10% for misclassifying nsRCC as sRCC, as depicted in Table 2.

**Table 2:** Overall performance of the LVQ classifier on the training, validation and test cohorts.

Cohort	Accuracy (%)	False positive rate (%)	False negative rate (%)	Positive predictive value (%)	Negative predictive value (%)
Training	93.75	6.25	0	88.89	100
Validation	83.33	16.67	0	75	100
Test	70	10	20	75	66.67

### sRCC vs. nsRCC mpMRI phenotypes

**Figure 5:**
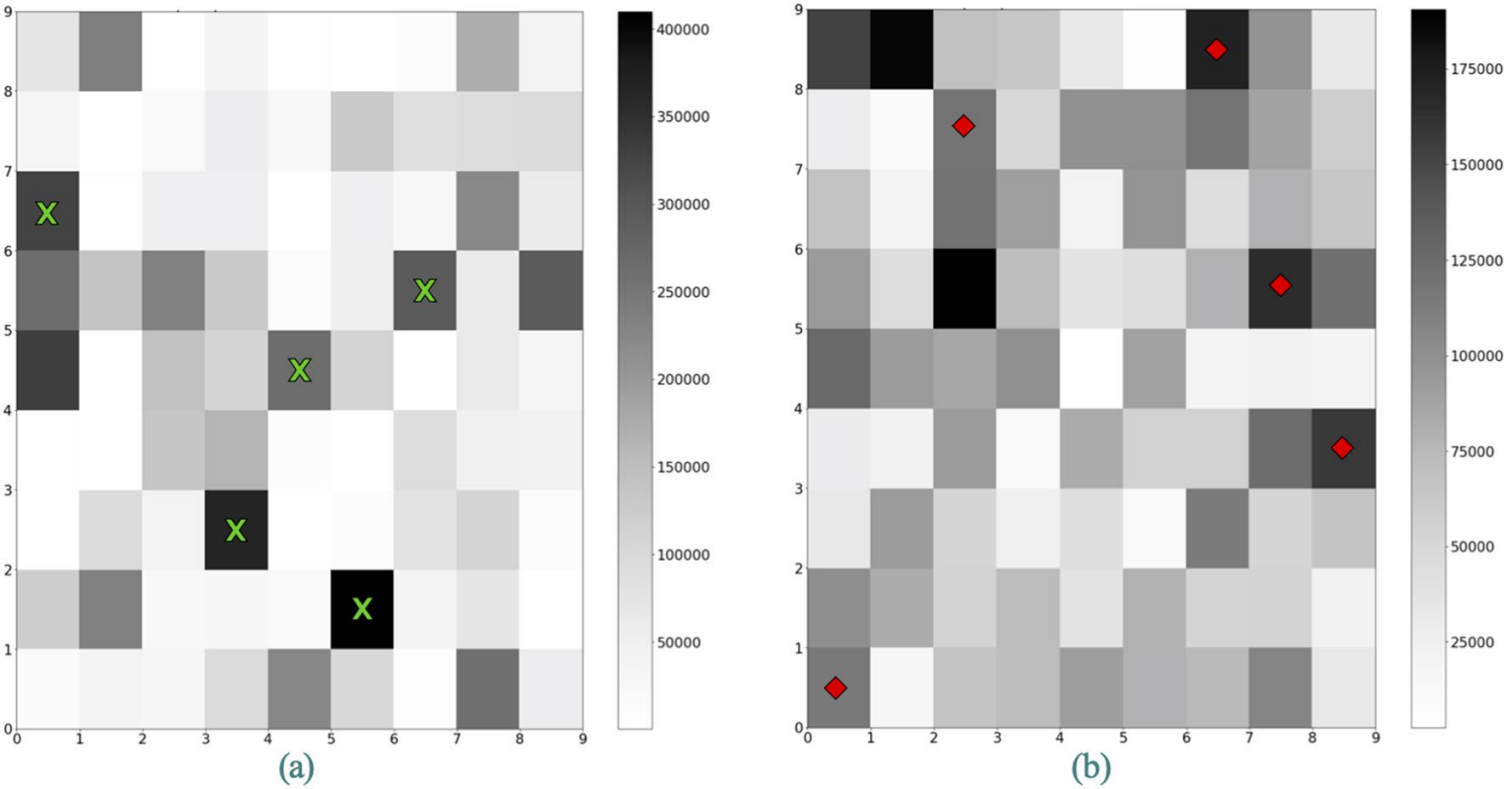
Activation Maps of voxels pooled from (**a**) all sRCC tumors, and, (**b**) all nsRCC tumors. The green X’s in (**a**) correspond to the five neurons with the greatest number of hits from sRCC tumors relative to hits from nsRCC tumors. The red diamonds in (**b**) correspond to the five neurons with the greatest number of hits from nsRCC tumors relative to hits from sRCC tumors.

An examination of the Activation Maps corresponding to the sarcomatoid and non-sarcomatoid tumors that are presented in Fig. 3 suggests some broad differences in the patterns of activations of the 81 neurons by tumors belonging to the two classes. To enhance these differences, in Fig. 5 we have shown two Activation Maps: one produced by analyzing pooled voxels from all sRCC tumors (Fig. 5a), and another produced from analysis of pooled voxels from all nsRCC tumors (Fig. 5b). The grayscale in Fig. 5a goes from zero hits in a neuron (white) to a maximum of 410,024 hits/neuron (black). The grayscale in Fig. 5b goes from zero hits in a neuron (white) to a maximum of 190,605 hits/neuron (black). The pooled Activation Map of nsRCC tumors has a “busier” appearance compared with the sparser pattern of activated neurons in the pooled Activation Map of sRCC tumors. All 81 neurons in each Activation Map would be analyzed by the LVQ. For illustrative purposes, we have marked on Fig. 5 the five neurons that were most frequently activated by voxels in sRCC tumors compared with nsRCC tumors (green X’s), and the five neurons that were most frequently activated by voxels in nsRCC tumors compared with sRCC tumors (red diamonds).

**Figure 6:**
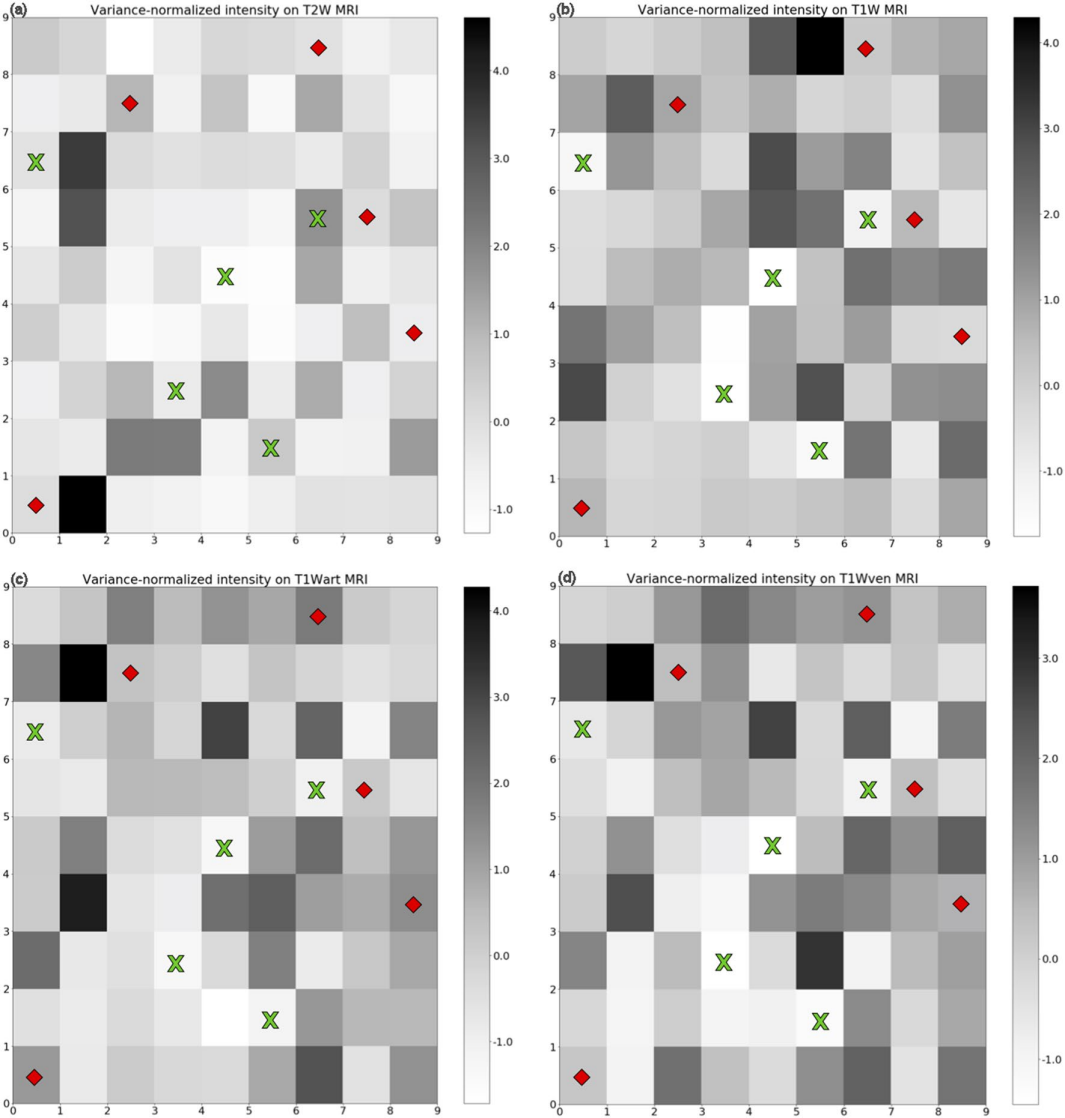
Calibrated and variance-normalized intensities on T2W, T1W, T1W-CEart and T1W-CEven images corresponding to each neuron. Zero values represent average intensity, negative values represent hypointensity (− 1 = one standard deviation below the mean), and positive values represent hyperintensity (+ 1 = one standard deviation above the mean).

To understand the mpMRI phenotypes underlying each neuron, the calibrated and variance-normalized intensities on T2W, T1W, T1W-CEart and T1W-CEven images that are associated with each neuron are depicted in Fig. 6. In each panel of Fig. 6, a grayscale value of zero corresponds to the average calibrated and normalized intensity computed from all tumor voxels in the pooled training and validation tumors on that particular MRI scan type. Negative values represent hypointensity, with a value of − 1 corresponding to an intensity that is one standard deviation below the mean computed from all tumor voxels in the pooled training and validation tumors. Positive values represent hyperintensity, with a value of + 1 corresponding to an intensity that is one standard deviation above the mean computed from all tumor voxels in the pooled training and validation tumors. Also marked in Fig. 6 are the 10 neurons from Fig. 5 that are among the most discriminative between the two classes of tumors.

We have listed the mpMRI characteristics of these 10 neurons in Table 3. Neurons 15, 22, 41, 55, and 52 are more representative of sRCC tumors than nsRCC tumors, and they are characterized by negative normalized intensity values (i.e., hypointensity) on T1W, T1W-art and T1W-del MRI. Neurons 1, 36, 53, 66 and 79, which are more representative of nsRCC tumors than sRCC tumors, are characterized by mostly positive normalized intensity values (i.e., hyperintensity) on T1W, T1W-art and T1W-del MRI.

**Table 3:** Calibrated and variance-normalized mpMRI intensities of neurons with the greatest differences in hits from sRCC vs. nsRCC tumors.

	Neuron #	T2W	T1W	T1Wart	T1Wven
sRCC–nsRCC	22	− 0.41	− 1.72	− 1.31	− 1.44
	15	0.60	− 1.43	− 1.15	− 1.25
	41	− 1.10	− 1.76	− 1.32	− 1.45
	55	− 0.12	− 1.36	− 0.84	− 0.61
	52	1.74	− 1.11	− 1.04	− 0.92
nsRCC–sRCC	79	− 0.09	0.17	1.83	1.16
	36	− 0.46	− 0.31	1.43	0.64
	53	0.07	0.55	0.18	0.40
	1	0.05	0.61	1.21	0.26
	66	1.04	0.92	0.36	0.43

## Discussion

### Activation maps as 2D fingerprints of mpMRI phenotypes

It is common to acquire multiparameter MRI images in several pathologies, especially renal cell cancer. We have presented an approach that combines image registration and pixel intensity calibration with two machine learning techniques, SOM and LVQ, that is useful for analyzing mpMRI datasets for the task of differential diagnosis. An intermediate step in this process is the reduction of multiple co-registered 3-dimensional mpMRI volumes to 2-dimensional “Activation Maps” (Fig. 3). Broadly speaking, Activation Maps of nsRCC tumors appear busier in comparison to Activation Maps of sRCC tumors, which look sparser. The potential for such visual pattern recognition on Activation Maps by a human expert, to augment the LVQ machine learning analysis of the Activation Maps, may make the process less of a “black box” and increase interpretability of the machine diagnosis. While the performance of our final model is not sufficient for immediate clinical utility, our results on an independent test dataset do point to the promise of the proposed approach with limited sample sizes. Deep learning algorithms such as CNN have been reported to achieve high accuracy on testing but require large data sets for model training ^7^.

### CT and MRI biomarkers for diagnosis of sRCC

Schieda et al. ^37^ investigated 10 sRCC and 12 non-sarcomatoid clear cell RCC on pre-operative triphasic renal CT that included images from unenhanced CT and the corticomedullary and nephrographic phases of contrast-enhanced CT. The CT images were assessed by two Radiologists for qualitative features that included tumor heterogeneity, tumor margin, calcification, intratumoral and peritumoral neovascularity, and invasion of the renal sinus, renal vein and adjacent organs. In this exploratory study they reported that a large tumor size, the presence of peritumoral neovascularity, and larger peritumoral vessels were features that are more commonly associated with sRCCs than with clear cell RCCs. On unenhanced CT images of 14 sRCC and 17 non-sarcomatoid clear cell RCC tumors they also computed texture features pertaining to gray-level co-occurrence and run-length matrix within manually defined regions-of-interest (ROIs) drawn on 3 selected slices per tumor. The extracted texture features were divided into subsets and used to train a SVM classifier as well as a logistic regression predictor. On the training dataset the SVM achieved an average accuracy of 55–68%, while the logistic regression model produced an accuracy of 55–81%.

mpMRI images have been reported to be informative for the task of identifying sRCC. In an analysis of 11 sRCC tumors, Takeuchi et al. ^38^ divided the intratumoral region into two regions, one in which pixels were hypointense on T2W relative to the contralateral renal cortex (T2LIA), and another in which pixels were iso- or hyper-intense on T2W relative to the contralateral renal cortex (T2HIA). They evaluated mean ADC and DCE-MRI signal intensity normalized to paraspinal muscle in these two intratumoral regions. Although they did not have access to whole mount post-resection specimens, they performed a radiologic-pathologic assessment of histopathologic specimens and hypothesized that the T2LIA regions corresponded to tumor areas with sarcomatoid differentiation. They proposed that the presence of regions in clear cell RCC tumors that were T2LIA with restricted ADC and low contrast enhancement on DCE-MRI might be characteristic of sRCC. In a follow-up study of 10 sRCC and 131 nsRCC tumors, Takeuchi et al. ^39^ analyzed the T2LIA content of tumors and their invasive nature on MRI images. Although their samples were unbalanced between sRCC and nsRCC, in a blinded assessment by two radiologists they achieved sensitivity, specificity and accuracy values of 90–95%, with a positive predictive value of 56% and a negative predictive value of 99%, for the task of diagnosing sRCC. Our findings (Table 3) are in agreement with a previous report by Takeuchi et al. ^38^ who noted that when these “T2LIA” regions in clear cell RCC tumors presented with low contrast enhancement on DCE-MRI it was more characteristic of sRCC than nsRCC. While this level of agreement between our findings and those of Takeuchi et al. is reassuring, it should be reiterated that all 81 neurons, representing 81 distinct combinations of calibrated intensities on the 4 mpMRI image types, inform the classification of individual tumor Activation Maps by the trained LVQ in our analysis.

### Study limitations

A strength of our study is that we have reported the performance of our classifier on unseen test data. A limitation of our study was the small sample size, though our sample size of 16 sRCC tumors is similar to those in radiologic studies published by other groups. This was a constraint imposed by the relatively low incidence of sRCC, and by our requirement for availability of pre-operative T2W, T1W, T1W-CEart, and T1W-CEven MRI images for each study subject. Additionally, based on the limited availability of sarcomatoid tumors at the time of data collection, the sRCC group had heterogeneous background subtypes which included mainly clear cell, but also chromophobe and papillary RCC. Future larger studies would benefit from examining sarcomatoid involvement arising within a single RCC subtype. In future work we will also include diffusion-weighted MRI in the SOM-LVQ analysis, as DW-MRI has been reported to be useful for this particular diagnostic problem ^38,39^. There is also scope for further improvements in model performance through optimization of the SOM order and tuning of other SOM-LVQ hyperparameters. We will also explore this SOM-LVQ method for other classification problems, such as for differential diagnosis of RCC subtypes on mpMRI. Additionally, future studies could evaluate the performance of the SOM-LVQ method in conjunction with other clinical and quantitative imaging measures that are suggestive of sRCC.

Another limitation of this initial work is that we have utilized manual contouring of tumors in our analysis. Kocak et al. have demonstrated that variability of manual delineation of RCC tumors on single 2D CT slices affects the reproducibility of radiomic features computed within the ROI ^40^ and the robustness of any diagnostic model built upon those features ^41^. In our current work we have sought to decrease variability stemming from ROI delineation by contouring both the tumor and the contralateral renal cortex on multiple slices rather than just a single 2D slice. Our analysis uses input data from multiple co-registered scans, and the goodness of co-registration between the T2W, T1W-unenhanced, T1W-CE-arterial, and T1W-CE-delayed images will also impact the robustness of our analysis. In this work we have used local registration around the tumor to improve alignment of voxels across the four MRI sequences, to reduce this potential source of variability. In future work we will systematically vary all three major interacting factors, namely co-registration between sequences, manual contouring of the tumor, and semi-automatic contouring of the renal cortex, to characterize the reproducibility and robustness of the final model prediction.

## Conclusions

We have demonstrated a Self-Organizing Map based approach for analysis of standard multiparametric MRI images to aid in the task of identifying sarcomatoid differentiation in renal cell carcinoma. Sarcomatoid differentiation is noted in approximately 5–10% of all RCCs, and this relatively uncommon presentation, combined with our requirement for the availability of pre-operative MRIs, restricted the current study to relatively small sample size. The performance of our final model on an independent test dataset, while leaving much room for improvement, points to the promise of this machine learning approach with limited sample sizes. The 2-dimensional “Activation Maps” that are produced as an intermediate output can be visually assessed by the human expert (i.e., Radiologist), which may increase interpretability and acceptability of the machine diagnosis. Our ongoing work is focused on increasing the sample size as well as on increasing the number of mpMRI “channels” to increase the information available to the SOM-LVQ model. Looking ahead, one can envision a hybrid diagnostic approach that combines the objective output of the LVQ classifier on the Activation Map of a given patient’s tumor, with a radiologist’s assessment of T2LIA content ^38,39^ and peritumoral vascular features ^37^, to achieve a combined power that is high enough to be clinically useful for diagnosis of sRCC on pre-operative mpMRI images.
